# Diagnostic Accuracy of Rapid Diagnostic Tests Versus Enzyme-Linked Immunosorbent Assay (ELISA) Across Major Infectious Diseases: A Systematic Review and Meta-Analysis

**DOI:** 10.7759/cureus.117107

**Published:** 2026-09-28

**Authors:** Priyanka Gupta, Saurabh Bijalwan, Deepjyoti Kalita, Garima Mittal

**Affiliations:** 1 Microbiology, Gautam Buddha Chikitsa Mahavidyalaya, Dr. K.K.B.M Subharti Hospital, Dehradun, IND; 2 Pharmacology, Gautam Buddha Chikitsa Mahavidyalaya, Dr. K.K.B.M Subharti Hospital, Dehradun, IND; 3 Microbiology, All India Institute of Medical Sciences, Guwahati, Guwahati, IND; 4 Microbiology, Himalayan Institute of Medical Sciences, Swami Rama Himalayan University, Dehradun, IND

**Keywords:** dengue, diagnostic accuracy, elisa, hepatitis b, hepatitis c, hiv, infectious diseases, meta-analysis, rapid diagnostic test, sensitivity

## Abstract

Rapid diagnostic tests (RDTs) are increasingly used for point-of-care diagnosis of infectious diseases because of their rapid turnaround time and ease of use. However, their diagnostic performance compared with enzyme-linked immunosorbent assay (ELISA) varies across diseases and assay platforms. This systematic review and meta-analysis was conducted according to PRISMA 2020 guidelines. A total of 26 studies were included in the qualitative synthesis, of which 13 datasets from 11 studies with complete 2 × 2 diagnostic data were included in the meta-analysis. Quality assessment was performed using QUADAS-2 (Quality Assessment of Diagnostic Accuracy Studies-2). Pooled diagnostic performance was summarized separately for each infection using extracted true-positive (TP), false-positive (FP), true-negative (TN), and false-negative (FN) values; forest plots, an HSROC (Hierarchical Summary Receiver Operating Characteristic model)-style summary visualization, and a Deeks-type funnel plot were generated. The aggregate diagnostic counts were TP = 1,304, FP = 68, TN = 24,301, and FN = 303. Overall, the included datasets showed consistently high specificity (≥96.97%), whereas sensitivity varied widely (25%-100%) across conditions. By target condition, the pooled sensitivity and specificity were human immunodeficiency virus (HIV; 78.6%/99.30%), hepatitis B virus (HBV; 88.2%/99.94%), hepatitis C virus (HCV; 75.7%/99.42%), dengue (97.0%/99.97%), and malaria (100.0%/97.18%). RDTs demonstrated high specificity and good sensitivity compared with ELISA, supporting their role as rapid screening tools, particularly in resource-limited settings. However, heterogeneity across diseases and assay platforms limits generalizability, and confirmatory testing remains important in selected clinical and blood-screening settings.

## Introduction and background

Rapid and accurate diagnosis of infectious diseases is essential for patient management, outbreak control, and interruption of transmission. Infections such as human immunodeficiency virus (HIV), hepatitis B virus (HBV), hepatitis C virus (HCV), malaria, and dengue continue to cause substantial morbidity, particularly in resource-limited regions. Early diagnosis allows timely treatment, appropriate counseling, infection-control measures, and rational use of confirmatory testing [1-4].

Rapid diagnostic tests (RDTs) are widely used as point-of-care tools because they are simple, inexpensive, and provide results within minutes. These advantages are important in emergency departments, peripheral laboratories, outreach camps, blood donor screening, and regions where laboratory infrastructure is limited [1,3,5]. Enzyme-linked immunosorbent assay (ELISA) remains a conventional laboratory-based method with strong analytical performance, but it requires equipment, trained personnel, batch processing, and a longer turnaround time [3,4].

Diagnostic performance of RDTs varies by infection, biological marker, stage of disease, assay design, sample type, and local epidemiology. HIV RDTs are generally highly accurate in validated algorithms, whereas sensitivity in HBV, HCV, dengue, and malaria may vary with antigen concentration, antibody kinetics, parasite density, day of illness, or test format [2,5-8].

Previous systematic reviews and meta-analyses have examined the diagnostic accuracy of RDTs for specific infectious diseases such as HIV, HBV, HCV, dengue, and malaria. However, these studies focused on individual diseases and did not provide an overall comparison of RDT performance against ELISA across multiple major infectious diseases. As a result, a broader understanding of how RDT accuracy varies across different infectious conditions remains limited.

To address this gap, the present review evaluated studies involving individuals tested for selected infectious diseases (population), using RDTs as the index test and ELISA as the reference standard, with sensitivity and specificity derived from 2 × 2 diagnostic data as the primary outcomes.

Accordingly, this systematic review and meta-analysis assessed the diagnostic accuracy of RDTs compared with ELISA across major infectious diseases. Using extracted 2 × 2 diagnostic data and QUADAS-2 (Quality Assessment of Diagnostic Accuracy Studies-2), the review aimed to synthesize disease-specific evidence on the sensitivity and specificity of RDTs and provide a cross-disease overview of their diagnostic utility.

## Review

Materials and methods

Study Design

This systematic review and meta-analysis was planned according to PRISMA 2020 guidelines (Figure 1) [9]. A protocol was developed prior to the conduct of the review but was not prospectively registered [10]. The review compared RDTs as index tests with ELISA as the comparator/reference laboratory assay across selected infectious diseases. Studies providing complete 2 × 2 contingency data were included in the quantitative meta-analysis.

**Figure 1:**
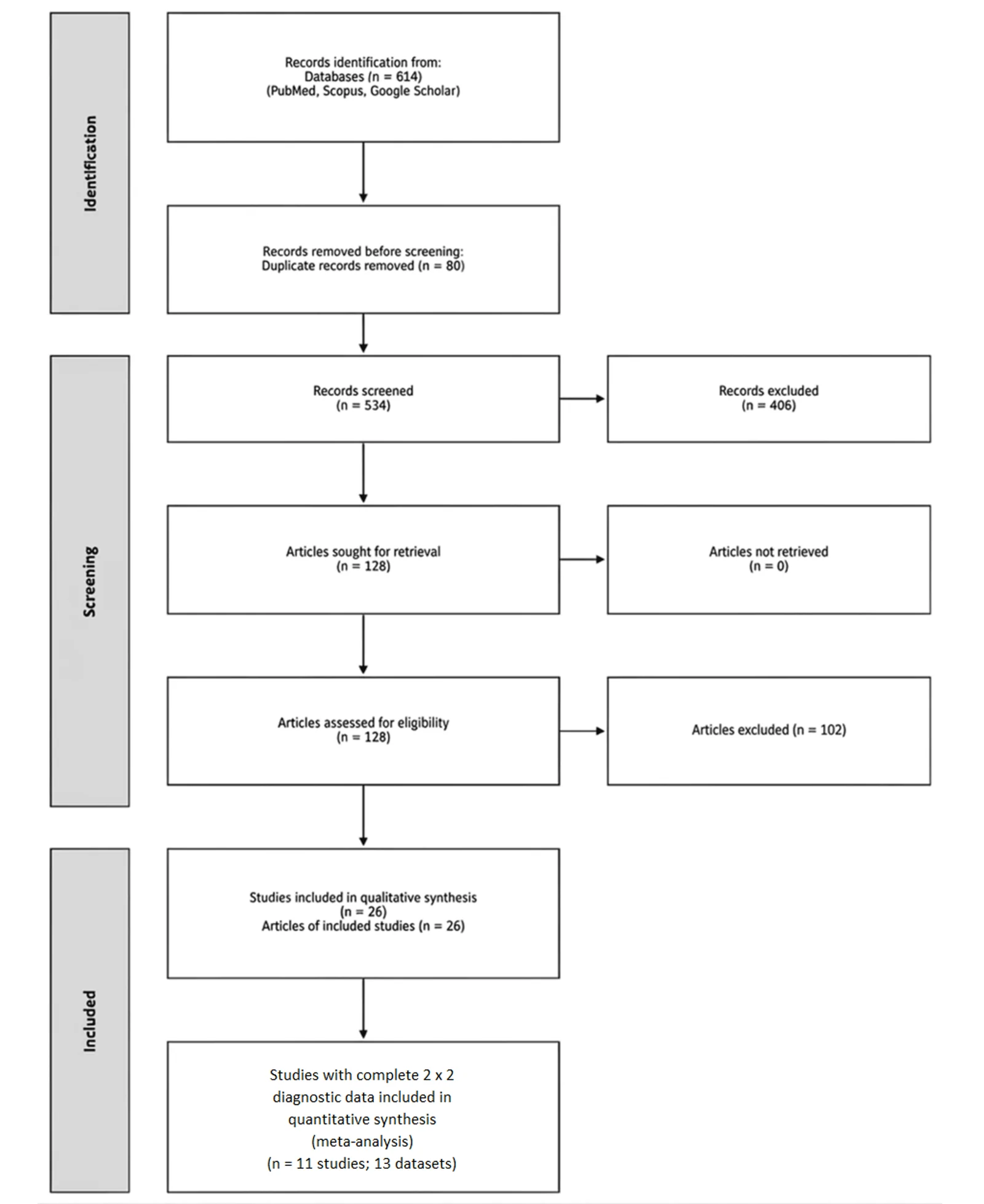
PRISMA flow diagram of study selection process

Search Strategy

A literature search was performed in PubMed, Scopus, and Google Scholar [11]. Search terms included combinations of "rapid diagnostic test," "RDT," "ELISA," "diagnostic accuracy," "sensitivity," "specificity," "HIV," "hepatitis B," "hepatitis C," "dengue," and "malaria." Reference lists of relevant articles were also screened. The literature search included studies available up to April 2026.

Eligibility Criteria

Studies were considered eligible if they: (1) involved human participants; (2) compared an RDT with ELISA for the diagnosis or screening of an infectious disease; and (3) reported sensitivity and specificity data or provided sufficient information to reconstruct true-positive (TP), false-positive (FP), true-negative (TN), and false-negative (FN) values. Case reports, narrative reviews, studies lacking a direct RDT-ELISA comparison, and studies comparing RDTs only with PCR, microscopy, culture, or clinical diagnosis were excluded from the quantitative meta-analysis.

Data Extraction

Extracted variables included author, year, country, disease, TPs, FPs, TNs, FNs, sensitivity, specificity, positive predictive value, and negative predictive value. When a single publication reported separate datasets for different infections, each dataset was entered separately. In a study by Preethi et al., the raw 2 × 2 values were used because the sensitivity and specificity percentages printed in the article were internally inconsistent with those counts [19].

Quality Assessment

Risk of bias and applicability concerns were independently assessed by two reviewers using the QUADAS-2 tool across the domains of patient selection, index test, reference standard, and flow and timing. Any disagreements were resolved through discussion and consensus [12-14].

Statistical Analysis

Sensitivity was calculated as TP/(TP + FN), specificity as TN/(TN + FP), positive predictive value as TP/(TP + FP), and negative predictive value as TN/(TN + FN). Wilson binomial intervals were used to calculate study-level confidence intervals. For each infectious disease, TP, FP, TN, and FN counts were aggregated across eligible datasets, and pooled sensitivity and specificity were calculated from these combined 2 × 2 counts. No overall pooled estimate across all diseases was generated because the included target conditions, biomarkers, and assay platforms were not considered clinically interchangeable.

HSROC (Hierarchical Summary Receiver Operating Characteristic model)-style scatter plots and Deeks-type funnel plot visualizations were generated using all 13 quantitative datasets. No formal HSROC or bivariate random-effects model was fitted because no single target condition contributed a sufficient number of datasets to support a reliable model. Similarly, no formal assessment of publication bias was performed because Deeks' test is generally recommended only when an adequate number of studies are available within the same target condition.

Heterogeneity was assessed descriptively through clinical and methodological characteristics of the included studies and by visual inspection of diagnostic performance across datasets. In accordance with guidance from the Cochrane Handbook for Diagnostic Test Accuracy (DTA) Reviews, I² and Cochran's Q statistics were not used because they were developed for intervention meta-analyses and do not adequately account for threshold effects or the correlation between sensitivity and specificity. Heterogeneity was therefore interpreted clinically rather than through a single summary statistic. In DTA reviews, heterogeneity is expected rather than a problem to be tested.

Results

The search identified 614 records. After removal of 80 duplicates, 534 records were screened. A total of 128 full-text articles were assessed, 26 studies were included in qualitative synthesis, and 13 different datasets from 11 studies with complete 2 × 2 diagnostic data were included in the quantitative meta-analysis (Figure 1). Table 1 demonstrates all the 13 datasets included in the present study [15-25]. Separate disease datasets (HBV, HCV, and HIV) were used from one publication (Al-Matary and Al Gashaa) and treated as separate datasets [18], while the study-level count remains one study.

**Table 1:** Summary of included studies with diagnostic 2 × 2 data TP: True positive; FP: False positive; TN: True negative; FN: False negative; PPV: Positive predictive value; NPV: Negative predictive value; HIV: Human immunodeficiency virus; HBV: Hepatitis B virus; HCV: Hepatitis C virus.

S. No.	Reference	Author	Year	Country	Disease	TP	FP	TN	FN	Sensitivity (%)	Specificity (%)	PPV/NPV (%)
1	[15]	Mehra et al.	2014	India	HIV	31	5	742	9	77.5	99.33	86.1/98.8
2	[16]	Goswami et al.	2016	India	HBV	70	0	10812	10	87.5	100	100.0/99.9
3	[17]	Kansra et al.	2021	India	HCV	789	0	1038	249	76.01	100	100.0/80.65
4	[18]-a	Al-Matary and Al Gashaa (HBV)	2022	Yemen	HBV	2	6	386	6	25	98.47	25.0/98.5
5	[18]-b	Al-Matary and Al Gashaa (HCV)	2022	Yemen	HCV	7	3	387	3	70	99.23	70.0/99.2
6	[18]-c	Al-Matary and Al Gashaa (HIV)	2022	Yemen	HIV	2	3	395	0	100	99.25	40.0/100.0
7	[19]	Preethi et al.	2023	India	HCV	2	2	64	2	50	96.97	50.0/97.0
8	[20]	Mandawat and Mathur	2019	India	HCV	6	0	181	1	85.7	100	100.0/99.5
9	[21]	Kalpana and Varma	2021	India	Dengue	198	0	2833	5	97.5	100	100.0/99.8
10	[22]	Naidu et al.	2022	India	Dengue	28	1	69	2	93.3	98.57	96.6/97.2
11	[23]	Haider et al.	2020	India	Malaria	75	2	69	0	100	97.18	97.4/100
12	[24]	Shrivastava and Chaurasia	2020	India	HBV	62	1	461	2	96.9	99.78	98.4/99.6
13	[25]	Solanke-Surase et al.	2026	India	HCV	32	45	6864	14	69.6	99.35	41.6/99.8

Specificity was consistently high (≥96.97), whereas sensitivity varied across conditions (25%-100%). The aggregate diagnostic counts were TP = 1,304, FP = 68, TN = 24,301, and FN = 303. Forest plots demonstrated that pooled sensitivity and specificity varied by target condition: HIV (78.6% and 99.30%); HBV (88.2% and 99.94%); HCV (75.7% and 99.42%); dengue (97.0% and 99.97%); and malaria (100.0% and 97.18%), respectively (Figures 2, 3). These results should be interpreted cautiously because the quantitative evidence was limited to 11 studies and included different infections, biomarkers, and assay platforms. Subtotals are aggregate estimates derived from pooled 2 × 2 counts within each condition; no overall cross-condition estimate is presented, as the tests and target conditions are not clinically interchangeable. The dataset by Preethi et al. contributed TP = 2, FP = 2, TN = 64, and FN = 2; these raw counts yield sensitivity and specificity of 50.0% and 96.97%, respectively [19].

**Figure 2:**
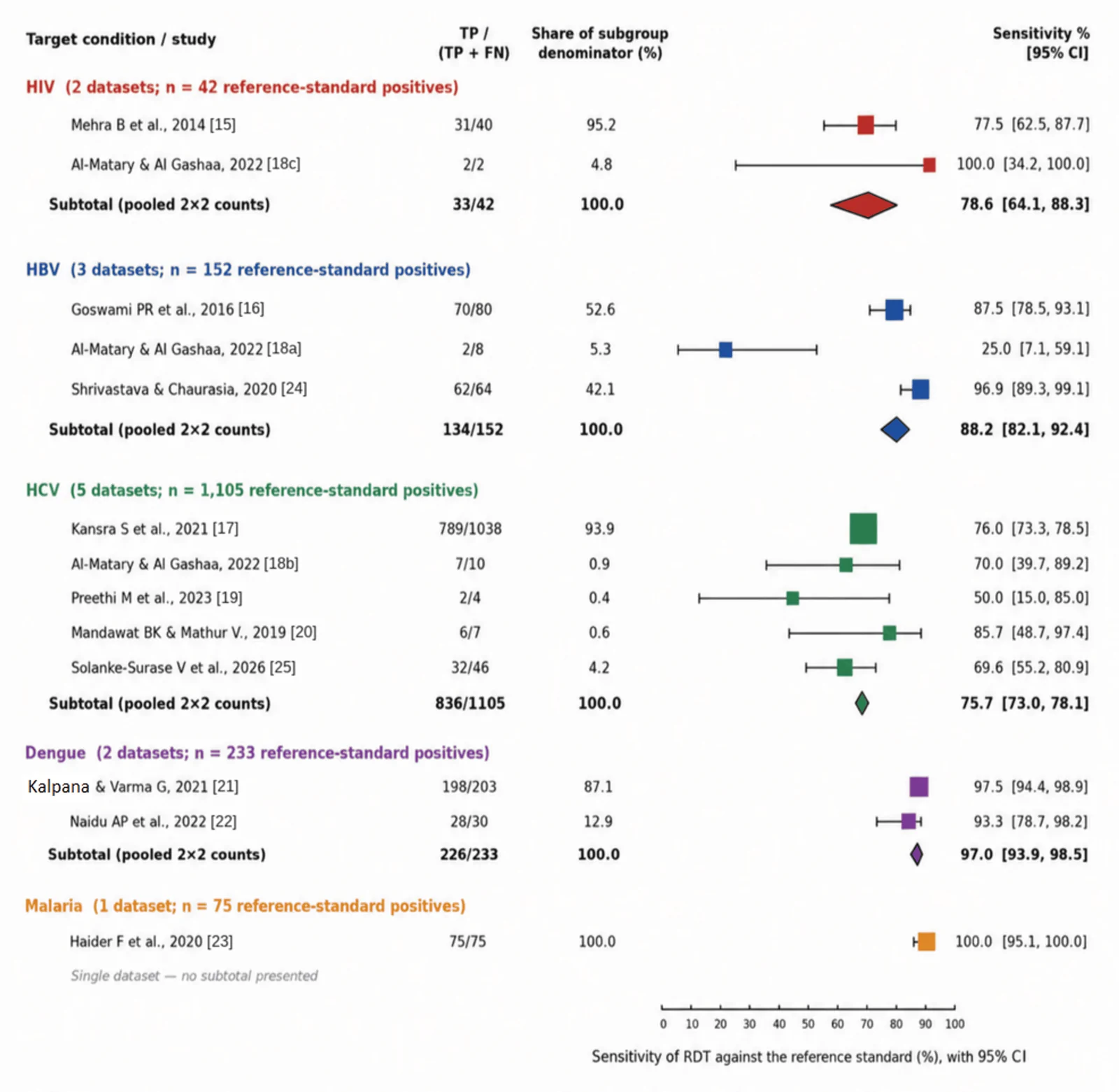
Forest plot of RDT sensitivity compared with ELISA Thirteen quantitative datasets from 11 studies; squares sized according to the number of reference-standard positives, with bars showing Wilson binomial 95% CIs. RDT: Rapid diagnostic test; ELISA: Enzyme-linked immunosorbent assay.

**Figure 3:**
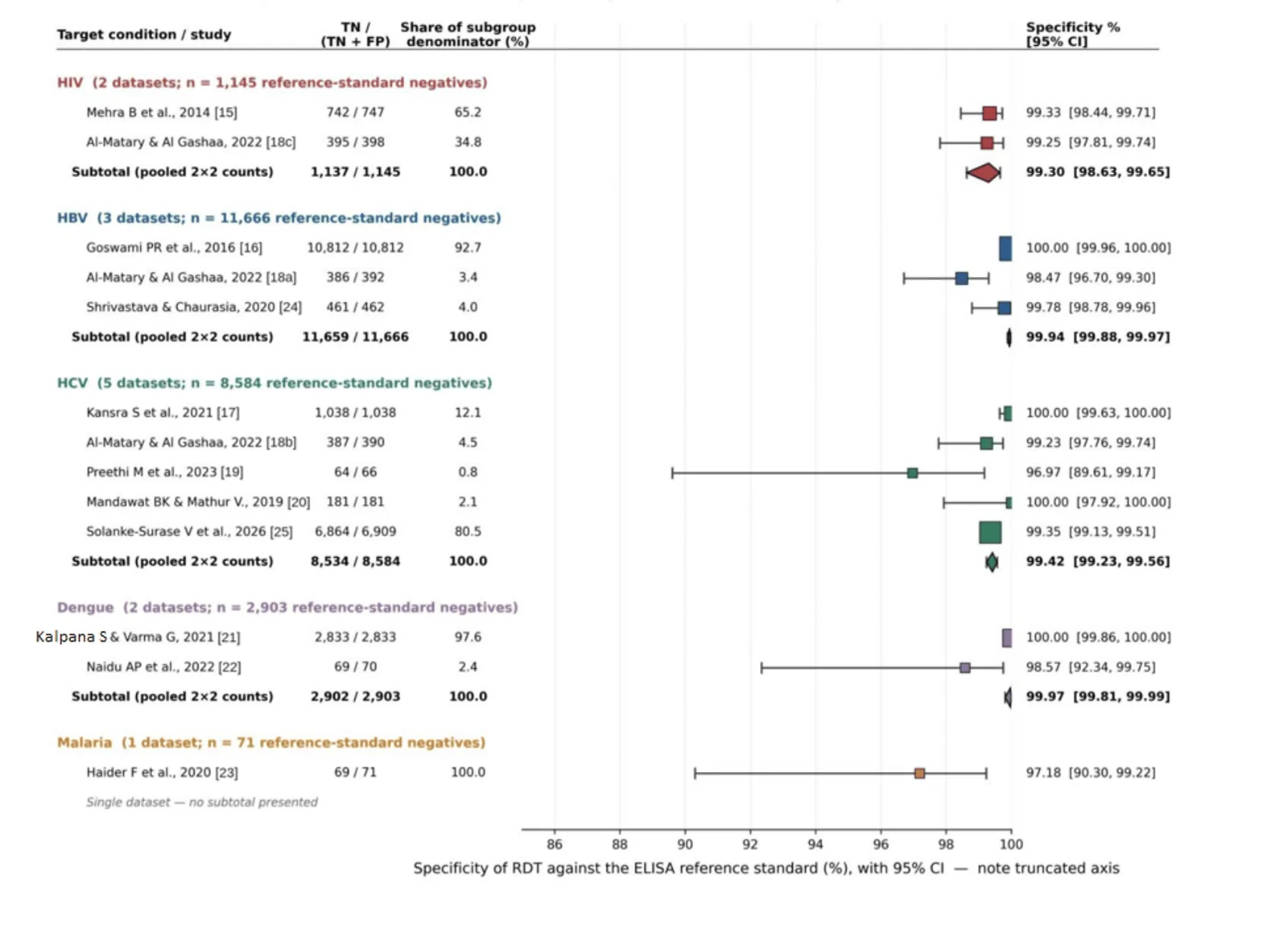
Forest plot of RDT specificity compared with ELISA Squares represent study-specific specificity estimates, with square size proportional to study weight. Horizontal lines indicate 95% confidence intervals calculated using Wilson binomial intervals. Diamonds represent pooled specificity estimates for disease-specific subgroups where two or more datasets were available. RDT: Rapid diagnostic test; ELISA: Enzyme-linked immunosorbent assay.

HSROC-Style Scatter Plot

Most datasets clustered in the upper-left of ROC space, reflecting high sensitivity and specificity, with two clear outliers-Preethi et al. (50.0% sensitivity) [19] and Al-Matary and Al Gashaa (HBV; 25.0% sensitivity) [18] (Figure 4, Panel B). No HSROC or bivariate summary curve was fitted, as no single target condition contributed enough datasets to support a fitted model (Figure 4, Panel A).

**Figure 4:**
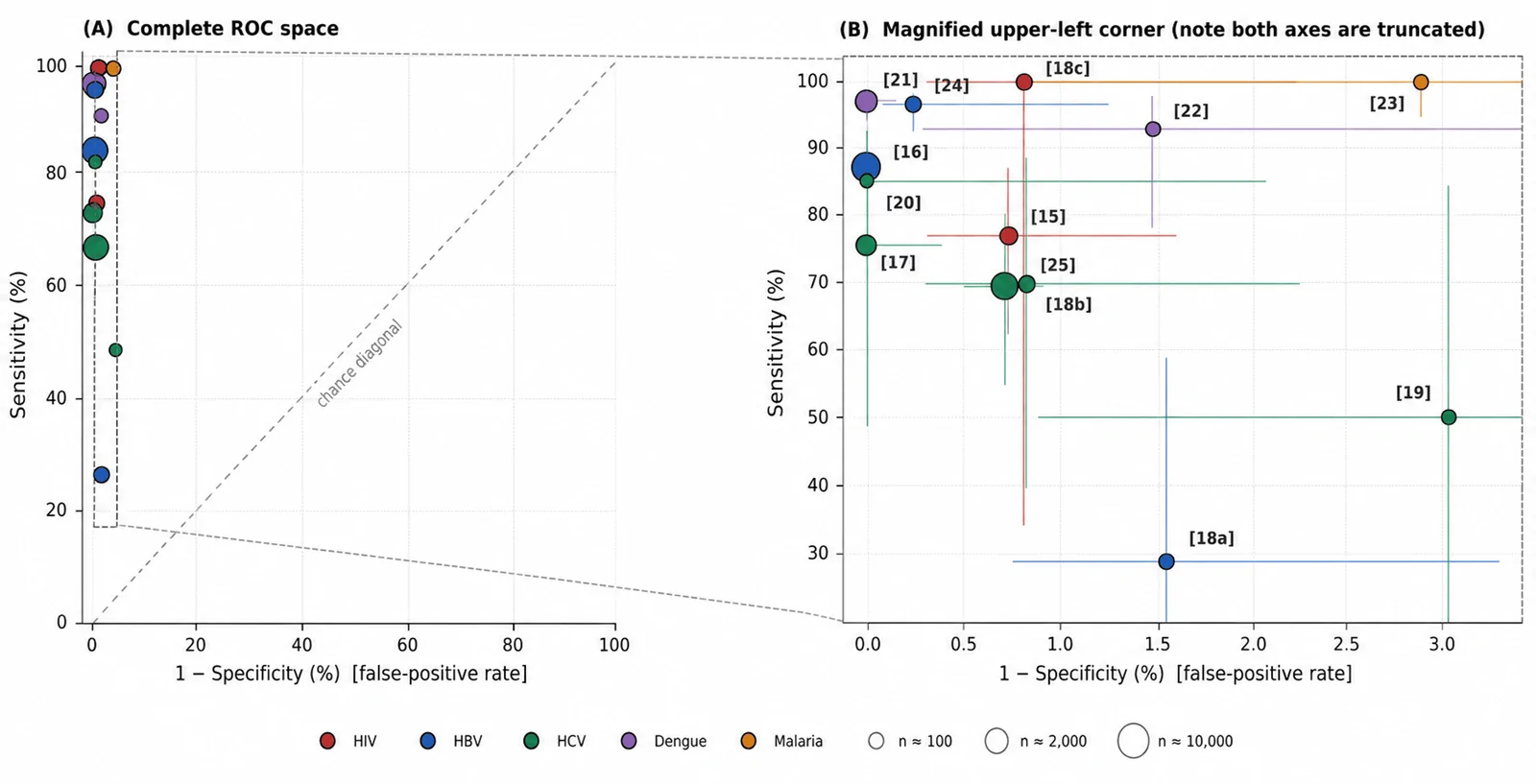
HSROC-style scatter plot of included quantitative datasets Thirteen quantitative datasets; circles sized according to total sample size, colored by target condition, and numbered according to the corresponding study listed in Table 1. Panel B additionally displays Wilson 95% confidence intervals for sensitivity (vertical bars) and 1 for specificity (horizontal bars). No HSROC curve was fitted because the datasets represented multiple diseases, with insufficient studies within individual disease groups to support hierarchical modeling. The spread of points reflects between-study heterogeneity, while clustering near the left margin indicates consistently high specificity. HSROC: Hierarchical Summary Receiver Operating Characteristic model. Sources: Refs. [15-25].

Deeks-Type Funnel Plot

This plot showed no clear asymmetry, though six datasets [16-18, 20, 21, 23] required a continuity correction for zero cells (Figure 5). No formal test was performed, as Deeks' test requires ≥ 10 studies within a single condition, so publication bias could not be confirmed or excluded (Figure 5).

**Figure 5:**
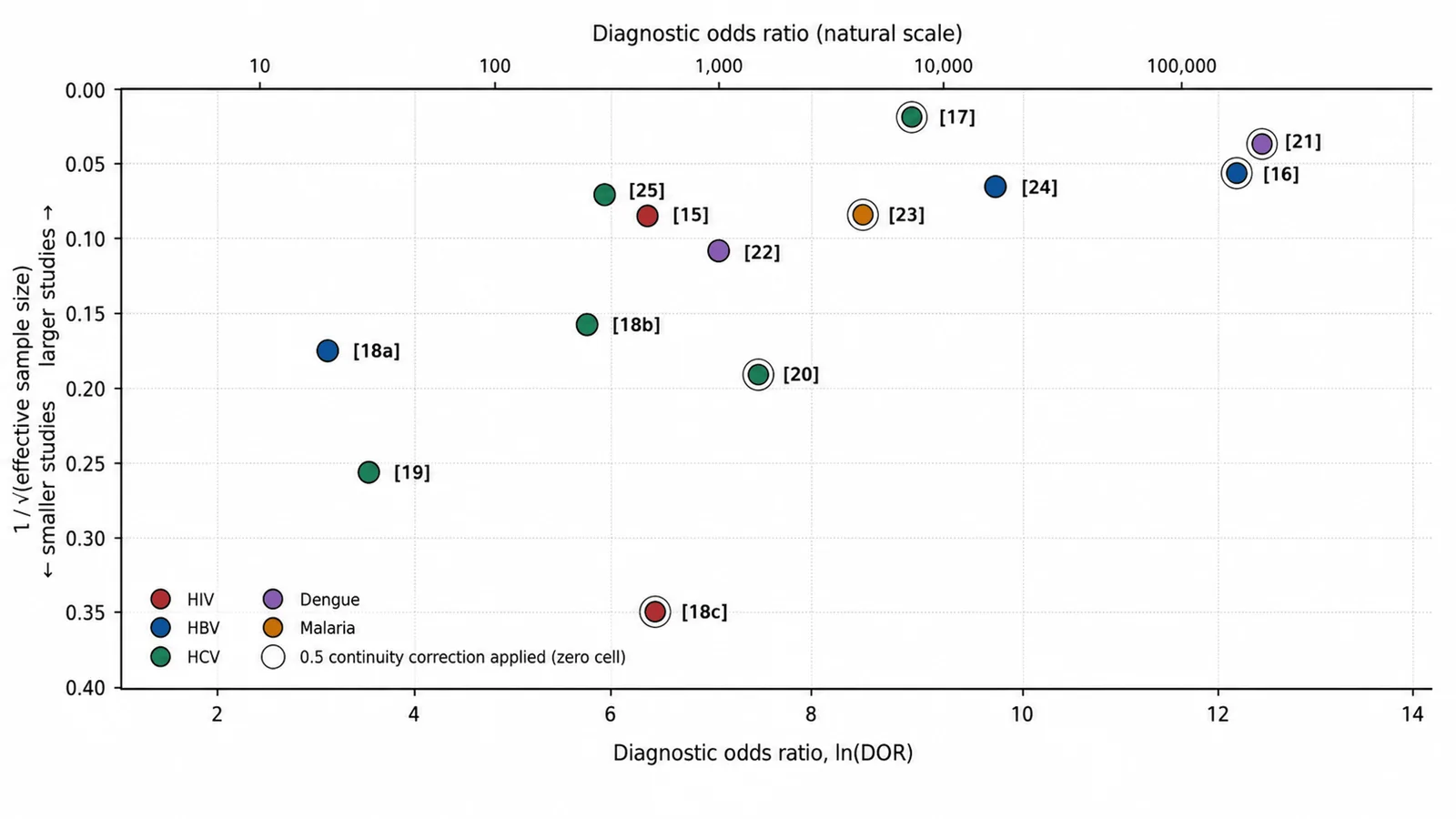
Deeks-type funnel plot for assessment of small-study effects Points are numbered according to Table 1 and colored by target condition. Ringed circles indicate datasets requiring a continuity correction. The figure shows a descriptive Deeks-type funnel plot. No formal Deeks regression test or regression line was performed because the datasets spanned multiple infectious diseases; therefore, publication bias could not be formally assessed. Sources: Refs. [15-25].

The QUADAS-2 assessment showed low risk of bias as the majority result across all domains (59%-79% of studies), with unclear risk in 10%-24% and high risk of bias in every domain (10%-17%), most notably in flow and timing (17%) and index test (11%) (Figure 6, Panel A). Applicability concerns were predominantly low across all three domains, with the reference standard showing the only instance of high concern (Figure 6, Panel B).

**Figure 6:**
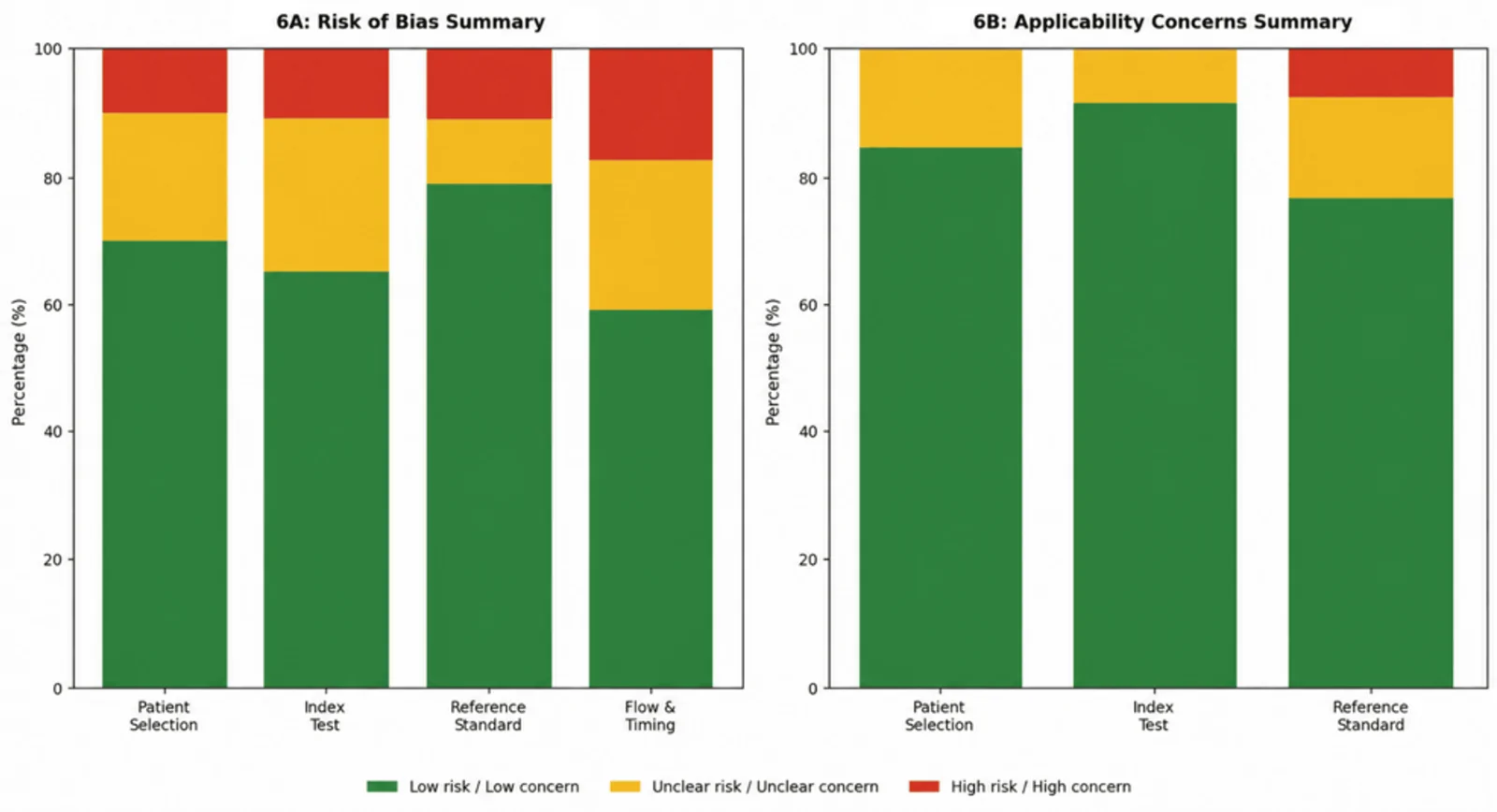
Methodological quality assessment using QUADAS-2 tool QUADAS-2: Quality Assessment of Diagnostic Accuracy Studies-2.

Discussion

This systematic review and meta-analysis evaluated the diagnostic accuracy of RDTs compared with ELISA across major infectious diseases. In general, RDTs showed high specificity (≥96.97), although sensitivity varied (25%-100%) between diseases. These findings should be interpreted cautiously given the limited quantitative evidence, heterogeneity in infections, biomarkers, and assay platforms. Accordingly, pooled estimates were considered within individual target conditions rather than a single cross-condition measure.

HIV

For HIV RDT, our findings (good pooled sensitivity of 78.6% and high pooled specificity of 99.30%) are comparable with the meta-analysis done by Kingwara et al. (2022) that found very high diagnostic accuracy for HIV RDTs (pooled sensitivity of 99% and specificity of 100%), especially in screening algorithms and decentralized settings [26]. The observed heterogeneity across studies likely reflects differences in kits, specimen type, and testing strategy, supporting algorithm-based rather than stand-alone interpretation.

HBV

For HBV RDT, our results (pooled sensitivity of 88.2% and pooled specificity of 99.94%) align with reviews showing high specificity (>98%) but variable sensitivity (around 88%-95%) for HBsAg RDTs compared with ELISA [27]. This suggests that positive RDT results are generally reliable, but negative tests should be interpreted cautiously in high-risk or clinically suspicious cases.

HCV

The present study found high specificity (>96%) but variable sensitivity (50%-85.7%) for HCV RDTs. Our pooled data show specificity of 99.42% and sensitivity of 75.7%, while the meta-analysis done by Tang et al. (2017) demonstrated excellent diagnostic performance of HCV antibody rapid diagnostic tests (pooled sensitivity and specificity of 98% and 100%) [28]. This discrepancy may reflect greater heterogeneity in the studies included in our meta-analysis, including differences in patient populations, assay performance, specimen types, and study settings. The evidence therefore supports RDTs as useful screening tools, but confirmatory testing remains important when clinical suspicion is high or when early infection is possible.

Dengue

For dengue, we found consistently high pooled sensitivity of 97.0% and pooled specificity of 99.97% with RDTs, which is comparable to the meta-analysis results by Mata et al. (2020) (NS1/IgM/IgG combination RDT: 91% sensitivity and 96% specificity). In contrast, Mata et al. (2020) also reported that IgM/IgG-only RDTs had poor sensitivity (~56%) [29].

This likely reflects differences in assay composition, sample timing, and study selection and suggests that the dengue RDTs included in our review were among the better-performing available tests. These findings support the use of combination RDTs for screening, while negative antibody-only RDT results may require confirmatory or repeat testing when suspicion remains high [30,31]. The reliability of our results for this particular infection is limited, as we could obtain data on only two studies.

Malaria

We could obtain results for only one study on malaria that demonstrated high specificity (97.18%) and sensitivity (100%). Large reviews on malaria found that RDT performance is influenced by parasite density, antigen variation, and persistent HRP2 antigenemia. This explains why malaria RDTs often show high specificity (96%-99%) but relatively variable sensitivity (89%-97%) in endemic settings, particularly when compared with laboratory-based reference methods [32,33]. The literature therefore supports their use as frontline diagnostic tools, while negative results in low parasite density or atypical infections should be interpreted with caution.

Cross-Disease Interpretation

The most consistent finding was high specificity (≥96.97). This suggests that positive RDT results are generally reliable in many screening settings. High specificity is particularly important in blood donor screening, outreach testing, and low-prevalence settings where FP results have clinical, psychological, and programmatic consequences.

Sensitivity showed greater variability (25%-100%). RDT performance depends on stage of infection, target antigen or antibody, analyte concentration, sample quality, and kit characteristics. A negative RDT result should therefore be interpreted cautiously when clinical suspicion is high.

The cross-disease HSROC-style pattern reinforces that RDT accuracy is context-dependent and should not replace disease-specific validation. The Deeks-type funnel plot was regenerated using all 13 quantitative datasets; nevertheless, assessment of small-study effects remains underpowered. From a practical perspective, based on all our results, the findings support a two-step diagnostic strategy: RDTs may be used for rapid initial screening, while ELISA or molecular tests remain important for confirmation, discordant results, blood screening, and clinically high-risk cases.

Strengths and Limitations

Strengths include PRISMA-guided study selection, extraction of complete 2 × 2 diagnostic data, inclusion of clinically relevant infectious diseases, QUADAS-2 quality assessment, and regeneration of all quantitative figures using the same 13 datasets.

Due to the limited datasets available for each target condition, formal hierarchical diagnostic meta-analysis models, such as bivariate random-effects or HSROC models, were not fitted. So, the reported pooled sensitivity and specificity values represent disease-specific summary measures derived from aggregated diagnostic counts and should not be interpreted as hierarchical pooled estimates. Other limitations include the small number of quantitative datasets, substantial cross-disease and assay heterogeneity, limited disease-specific subgroup analysis, review protocol development, but no prospective registration, and underpowered assessment of small-study effects (Deeks’ test). Deeks' funnel plot is recommended when ≥10 studies of the same target condition are available. So, we presented the funnel purely descriptively with no test statistic. The study by Preethi et al. contained sensitivity and specificity percentages that were inconsistent with those calculated from its published raw 2 × 2 table; therefore, the raw counts were used.

## Conclusions

RDTs demonstrated high specificity and good sensitivity compared with ELISA across major infectious diseases, supporting their utility as rapid screening tools. However, variability in sensitivity across diseases and assay platforms necessitates cautious interpretation, and confirmatory testing with ELISA or molecular methods remains important in selected clinical settings, discordant results, blood screening, and high-risk situations.

## Appendices

**Table 2:** PRISMA 2020 checklist for diagnostic accuracy of rapid diagnostic tests versus ELISA across major infectious diseases: a systematic review and meta-analysis Source: Ref. [9]. For more information, visit www.prisma-statement.org. ELISA: Enzyme-linked immunosorbent assay.

Topic	No.	Item	Location where item is reported
Title			
Title	1	Identify the report as a systematic review.	Title
Abstract			
Abstract	2	See the PRISMA 2020 for Abstracts checklist	
Introduction			
Rationale	3	Describe the rationale for the review in the context of existing knowledge.	Introduction and background
Objectives	4	Provide an explicit statement of the objective(s) or question(s) the review addresses.	Introduction and background
Methods			
Eligibility criteria	5	Specify the inclusion and exclusion criteria for the review and how studies were grouped for the syntheses.	Materials and methods - eligibility criteria
Information sources	6	Specify all databases, registers, websites, organizations, reference lists, and other sources searched or consulted to identify studies. Specify the date when each source was last searched or consulted.	Materials and methods - search strategy
Search strategy	7	Present the full search strategies for all databases, registers and websites, including any filters and limits used.	Materials and methods - search strategy
Selection process	8	Specify the methods used to determine whether a study met the inclusion criteria of the review, including the number of reviewers who screened each record and each report retrieved, whether they worked independently, and, if applicable, details of any automation tools used in the process.	Materials and methods - study design and search strategy
Data collection process	9	Specify the methods used to collect data from reports, including the number of reviewers who collected data from each report, whether they worked independently, any processes for obtaining or confirming data from study investigators, and, if applicable, details of automation tools used in the process.	Materials and methods - data extraction
Data items	10a	List and define all outcomes for which data were sought. Specify whether all results that were compatible with each outcome domain in each study were sought (e.g., for all measures, time points, and analyses) and, if not, the methods used to decide which results to collect.	Materials and methods - data extraction
	10b	List and define all other variables for which data were sought (e.g., participant and intervention characteristics and funding sources). Describe any assumptions made about any missing or unclear information.	Materials and methods - data extraction
Study risk of bias assessment	11	Specify the methods used to assess risk of bias in the included studies, including details of the tool(s) used, the number of reviewers who assessed each study and whether they worked independently, and, if applicable, details of automation tools used in the process.	Materials and methods - quality assessment
Effect measures	12	Specify for each outcome the effect measure(s) (e.g., risk ratio and mean difference) used in the synthesis or presentation of results.	Materials and methods - statistical analysis
Synthesis methods	13a	Describe the processes used to decide which studies were eligible for each synthesis (e.g., tabulating the study intervention characteristics and comparing against the planned groups for each synthesis (item 5)).	Materials and methods - eligibility criteria
	13b	Describe any methods required to prepare the data for presentation or synthesis, such as handling of missing summary statistics, or data conversions.	Materials and methods - data extraction
	13c	Describe any methods used to tabulate or visually display results of individual studies and syntheses.	Materials and methods - statistical analysis
	13d	Describe any methods used to synthesize results and provide a rationale for the choice(s). If meta-analysis was performed, describe the model(s), method(s) to identify the presence and extent of statistical heterogeneity, and software package(s) used.	Materials and methods - statistical analysis
	13e	Describe any methods used to explore possible causes of heterogeneity among study results (e.g., subgroup analysis and meta-regression).	Materials and methods - statistical analysis
	13f	Describe any sensitivity analyses conducted to assess robustness of the synthesized results.	Not done
Reporting bias assessment	14	Describe any methods used to assess risk of bias due to missing results in a synthesis (arising from reporting biases).	Materials and methods - quality assessment
Certainty assessment	15	Describe any methods used to assess certainty (or confidence) in the body of evidence for an outcome.	Materials and methods - statistical analysis
Results			
Study selection	16a	Describe the results of the search and selection process from the number of records identified in the search to the number of studies included in the review, ideally using a flow diagram.	Figure *1*
	16b	Cite studies that appeared to meet the inclusion criteria but were excluded, and explain why they were excluded.	N/A
Study characteristics	17	Cite each included study and present its characteristics.	Table *1*
Risk of bias in studies	18	Present risk-of-bias assessments for each included study.	Figure *6*
Results of individual studies	19	For all outcomes, present, for each study: (a) summary statistics for each group (where appropriate) and (b) an effect estimate and its precision (e.g., confidence/credible interval), ideally using structured tables or plots.	Figures *2*, 3
Results of syntheses	20a	For each synthesis, briefly summarize the characteristics and risk of bias among contributing studies.	Results
	20b	Present results of all statistical syntheses conducted. If meta-analysis was done, present for each the summary estimate and its precision (e.g., confidence/credible interval) and measures of statistical heterogeneity. If comparing groups, describe the direction of the effect.	Figures *2*, *4*
	20c	Present results of all investigations of possible causes of heterogeneity among study results.	Figures *4*, *5*
	20d	Present results of all sensitivity analyses conducted to assess the robustness of the synthesized results.	N/A
Reporting biases	21	Present assessments of risk of bias due to missing results (arising from reporting biases) for each synthesis assessed.	Figure *6*
Certainty of evidence	22	Present assessments of certainty (or confidence) in the body of evidence for each outcome assessed.	N/A
Discussion			
Discussion	23a	Provide a general interpretation of the results in the context of other evidence.	Discussion
	23b	Discuss any limitations of the evidence included in the review.	Strengths and limitations
	23c	Discuss any limitations of the review processes used.	Strengths and limitations
	23d	Discuss implications of the results for practice, policy, and future research.	Cross-disease interpretation
Other information			
Registration and protocol	24a	Provide registration information for the review, including register name and registration number, or state that the review was not registered.	Material and methods - study design
	24b	Indicate where the review protocol can be accessed, or state that a protocol was not prepared.	Material and methods - study design
	24c	Describe and explain any amendments to information provided at registration or in the protocol.	N/A
Support	25	Describe sources of financial or non-financial support for the review and the role of the funders or sponsors in the review.	Funding
Competing interests	26	Declare any competing interests of review authors.	Conflicts of interest
Availability of data, code, and other materials	27	Report which of the following are publicly available and where they can be found: template data collection forms; data extracted from included studies; data used for all analyses; analytic code; any other materials used in the review.	N/A
PRIMSA Abstract Checklist			
Topic	No.	Item	Reported?
Title			
Title	1	Identify the report as a systematic review.	Yes
Background			
Objectives	2	Provide an explicit statement of the main objective(s) or question(s) the review addresses.	Yes
Methods			
Eligibility criteria	3	Specify the inclusion and exclusion criteria for the review.	Yes
Information sources	4	Specify the information sources (e.g. databases and registers) used to identify studies and the date when each was last searched.	No
Risk of bias	5	Specify the methods used to assess risk of bias in the included studies.	Yes
Synthesis of results	6	Specify the methods used to present and synthesize results.	Yes
Results			
Included studies	7	Give the total number of included studies and participants and summarize relevant characteristics of studies.	Yes
Synthesis of results	8	Present results for main outcomes, preferably indicating the number of included studies and participants for each. If meta-analysis was done, report the summary estimate and confidence/credible interval. If comparing groups, indicate the direction of the effect (i.e., which group is favored).	Yes
Discussion			
Limitations of evidence	9	Provide a brief summary of the limitations of the evidence included in the review (e.g., study risk of bias, inconsistency, and imprecision).	Yes
Interpretation	10	Provide a general interpretation of the results and important implications.	Yes
Other			
Funding	11	Specify the primary source of funding for the review.	No
Registration	12	Provide the register name and registration number.	No
